# The environmental externalities of tobacco manufacturing: A review of tobacco industry reporting

**DOI:** 10.1007/s13280-019-01148-3

**Published:** 2019-03-09

**Authors:** Yogi Hale Hendlin, Stella A. Bialous

**Affiliations:** 1 Erasmus School of Philosophy, Dynamics of Inclusive Prosperity Initiative, Erasmus University Rotterdam, Rotterdam, The Netherlands; 2 Environmental Health Initiative, University of California San Francisco, 530 Parnassus Avenue, Suite 366, San Francisco, 94143 USA; 3 Center for Tobacco Control Research and Education, School of Nursing, University of California San Francisco, 530 Parnassus Avenue, Suite 366, San Francisco, 94143 USA

**Keywords:** Drivers of climate change, Industrial externalities, Product manufacturing, Sustainability, Tobacco industry

## Abstract

Growing research and public awareness of the environmental impacts of tobacco present an opportunity for environmental science and public health to work together. Various United Nations agencies share interests in mitigating the environmental costs of tobacco. Since 2000, transnational tobacco industry consolidation has accelerated, spotlighting the specific companies responsible for the environmental and human harms along the tobacco production chain. Simultaneously, corporate social responsibility norms have led the industry to disclose statistics on the environmental harms their business causes. Yet, independent and consistent reporting remain hurdles to accurately assessing tobacco’s environmental impact. This article is the first to analyze publicly available industry data on tobacco manufacturing pollution. Tobacco’s significant environmental impact suggests this industry should be included in environmental analyses as a driver of environmental degradation influencing climate change. Countries aiming to meet UN Sustainable Development Goals must act to reduce environmental harms caused by the tobacco industry.

## Introduction

The World Health Organization’s (WHO) 2017 report “Tobacco and Its Environmental Impact: An Overview” calls attention to the environmental burden of growing, curing, packaging, transporting, manufacturing, and distributing 6.25 trillion cigarette sticks annually (Hendlin 2017; Kelly 2017; World Health Organization 2017a). So far, the global tobacco control agenda has mainly focused on the one billion smokers and seven million people per year dying globally from tobacco use and exposure (Ng et al. 2014; Novotny et al. 2015; Reitsma et al. 2017; World Health Organization 2017b). Yet, important research examining deforestation (Otañez et al. 2009; Otañez and Glantz 2011; Eriksen et al. 2015) and cigarette butt waste (Novotny et al. 2009; Healton et al. 2011; Slaughter et al. 2011; Curtis et al. 2014) has made the public health case for confronting tobacco’s environmental impact—creating allies between public health and environmental interests (Freiberg 2014; Curtis et al. 2016; Wallbank et al. 2017).

Tobacco smoke emissions from cigarettes alone on a global scale contribute significant masses of toxicants to the global environment. In a single year, direct emissions from smoking contribute tens of thousands of metric tons of known human carcinogens, toxicants, and greenhouse gases (Repace 2004). Toxic emissions from all smoked cigarettes annually include an estimated 3000–6000 metric tons of formaldehyde and 12 000–47 000 tons of nicotine (Novotny et al. 2015). In addition, three major greenhouse gases are released in significant amounts via tobacco smoke: carbon dioxide, methane, and nitrous oxides (Gilmour et al. 2006; World Health Organization 2017a). One study found that the environmental pollution from smoking three cigarettes caused up to ten times the small particulate matter (PM_2.5_) concentrations of idling a diesel car engine for 30 minutes (Invernizzi et al. 2004).

Hand in hand with tobacco’s ecological harms, the environmental justice consequences of tobacco are pressing. The human harms from deforestation (Lecours et al. 2012; Leppan et al. 2014; Jew et al. 2017; Jimu et al. 2017), farm workers suffering from green leaf sickness (Schep et al. 2009; Benson 2011; Faria et al. 2014; Bonamonte et al. 2016), soil exhaustion (Leppan et al. 2014), and other fallout from tobacco farming, mostly occurring in low- and middle-income countries, has become legible to environmental organizations, governments, and intergovernmental institutions. The sizable environmental impact of cigarette butt litter—the most pervasive litter item found on beach clean ups (Novotny et al. 2009; Novotny and Slaughter 2014)—also pollutes terrestrial and aquatic ecosystems (Healton et al. 2011; Slaughter et al. 2011).

Industrial ecology research on product lifecycle analysis, however, has yet to adequately address the tobacco industry’s considerable contribution to environmental pollution and degradation (Trucost 2009).

The United Nations’ Sustainable Development Goals (SDGs) which aim to address global challenges to both sustainability and development (United Nations 2015a), incorporate as target 3a the WHO Framework Convention on Tobacco Control (FCTC) (World Health Organization 2003; United Nations 2015a, b), the landmark international treaty and global governance structure ratified by 181 countries to reduce the damage of the tobacco epidemic. This reinforces the need to focus on tobacco as a global industry considerably contributing to environmental degradation and health inequalities (Kulik et al. 2017). Increasingly, the reinforcing effects of environmental sustainability and public health dovetail in their fight to reduce the consequences of tobacco.

This article is the first to review the environmental costs of tobacco manufacturing with the industry’s own published data. While estimations exist (Zafeiridou et al. 2018), quantifying the environmental damage of the tobacco industry has not yet been fully measured, understood, or acted upon. This is due in part to a lack of accurate, reliable, independent environmental reporting and data transparency. Until the early 2000’s, few data were publicly available. Since the early 2000’s, some data has been made voluntarily available through pressure on the industry to abide by prevailing corporate social responsibility (CSR) standards, although these reports are neither systematic nor standardized. Nonetheless, analyzing the tobacco industry’s own data reporting the environmental costs of tobacco manufacturing clarifies the contribution of tobacco to environmental pollution, even if this self-reported data emerges from flawed methods and an overly narrow scope.

## Methods

Our analysis proceeds from the self-reported industry data published in public documents. From January through May 2018 we examined tobacco industry sustainability reports and annual investor reports from 2005 to 2018, as well as UN Global Compact reports (before tobacco companies were excluded from this organization in September 2017), Carbon Disclosure Project reports, and other publicly available resources to gather industry-reported data on the environmental costs of tobacco manufacturing. Although we reviewed data since 2005, whenever possible, we used the most updated environmental reporting information available through July 2018. We also drew upon previous estimates of the environmental costs of tobacco in the peer-review literature and third-party reports.

The largest tobacco companies currently report their annual energy use, CO_2_-equivalent emissions, water use, water discharge, hazardous waste, and total waste, including or omitting different areas of reporting over time. For example, Altria does not report water discharge data, Japan Tobacco International (JTI) stopped after 2014 reporting intensity (number of cigarettes produced or millions of dollars of revenue per unit of pollution), and the granularity of reporting detail differs dramatically by corporation. To the extent possible, current data on these metrics is included here for six major tobacco companies: Altria/Philip Morris, Philip Morris International (PMI), Reynolds American Inc. (RAI, now a subsidiary of BAT), British American Tobacco (BAT), Imperial Brands (formerly Imperial Tobacco), and Japan Tobacco International (JTI). China National Tobacco Company (CNTC) is addressed separately, as the company, which produces more than 40% of the world’s cigarettes, appears to follow different voluntary reporting systems. Available manufacturing data pertain mainly to cigarettes, rather than to smokeless tobacco or electronic-cigarettes (e-cigarettes).

## Theoretical framework and background

While the questions surrounding the tobacco industry’s corporate social responsibility (CSR) reporting and auditing are unique due to the scrutiny this particular industry receives, the problems of industry externalities and the lack of transparent third-party auditing are more general problems with the CSR paradigm shared by other companies (Tesler and Malone 2008; Hirshbein 2012; Fooks et al. 2013; McDaniel et al. 2016, 2018). Fernando and Lawrence (2014) propose an integrated theoretical framework for explaining CSR practices by bringing together three inter-related and complementary theories: legitimacy theory, stakeholder theory and institutional theory. This integrated approach supports the existing research on the motivation and impact of the tobacco industry’s own CSR efforts. While tobacco CSR programs and the marketing of these programs is constrained more than some industries (Dorfman et al. 2012; McDaniel and Malone 2012), insofar as in many developed countries they cannot use their CSR donations to explicitly promote their product to youth, nonetheless like other industries the tobacco industry seeks to profit from their CSR efforts and “neutralize” negative publicity (Gonzalez et al. 2012; Fooks et al. 2013). Our results are discussed through understanding that the tobacco industry’s efforts to reduce their environmental harms amount to CSR initiatives displaying a lack of transparency and independent verification, that limit objective assessment of the environmental impact of tobacco manufacturing.

Accounting for the environmental impact of tobacco manufacturing requires foremost having access to reliable data. Two problems arise: one procedural, the other epistemological. While environmental accounting in the last decades has become less haphazard and more scientific, it remains an inexact art. Open questions include: do consulting and auditing firms have full access to industry data, and is the industry reporting everything? Are companies aware of all externalities, or may there be other costs not yet reckoned due to conceptual blinders? Are these data being fully reported? From the tobacco industry’s publicly available materials, there are clear gaps and discrepancies from year to year. If the data exist, why are they not reported? If they do not exist, why not?

The lack of independent third-party oversight of these reports, i.e., oversight from agencies not directly paid and thus incentivized by tobacco company interests to favorably report, also is common among many industries, not just tobacco (Fooks et al. 2013). CSR “disclosure interaction effects” may take place if there is incentive on the part of management to deliver CSR goals, undermining the reliability of assurance agency reports for both investors and the public (Brown-Liburd and Zamora 2015). This problem exists across many industries. Although the Global Reporting Initiative aims to develop standards for CSR auditing, because CSR assurance companies operate in a “competitive, mainly unregulated market,” the credibility of directly industry-paid CSR assurances can lack, or be perceived to lack, credibility (Cohen and Simnett 2015).

Because tobacco’s particular harm to human and environmental health, and the non-essential status of the product, mandating data transparency for tobacco manufacturing warrants prioritization. Policies to provide a mechanism for outside accounting could consider tobacco product taxes to account for environmental impact, and then allow independent auditing of the tobacco industry using state funds, creating a financial firewall between industry and CSR assurance agencies. Currently, however, such an arrangement is absent. Piecemeal rather than organized reporting, and in-house rather than government or agency oversight on environmental pollution, greatly restrict current scientific assessments of the environmental impacts of tobacco product manufacture (Hirschhorn 2004; Moerman and Van Der Laan 2005; Palazzo and Richter 2005; Fooks et al. 2011). Stipulating a standardized metric, assessed by disinterested third-party reporting agencies would be a first step to accurately determine the true costs of tobacco production.

Research on what motivates industries to respond to their environmental and social impact exists for many industries, not just tobacco. Companies tend to act based on a mixture of novel policy constraints, updated risk assessments, cost offsets, and the business opportunities that arise in tackling externalities (Agrawala et al. 2011; Glaas et al. 2017). Brand image is also crucial to a CSR calculus (McDaniel and Malone 2009; Hastings 2012). Some companies have been shown to spend more money on *advertising* their CSR than they actually spent on sustainability or social responsibility projects (Gonzalez et al. 2012; Hastings 2012; McDaniel et al. 2018). Minimizing environmental harms through comparison with other industries is also a common tactic. For example, in PMI’s 2016 “Communication on Progress” for the UN Global Compact, PMI minimizes the water costs of tobacco, arguing that “[t]obacco growing and manufacturing take around one-third of the water required to make the same amount of tea or one-sixth that of coffee or chocolate (per weight of finished product)” (Philip Morris International 2016). PMI’s comparison attempts to put tobacco on par with these other products, ignoring the differentiator that these other products do not kill one in two of their daily users, as tobacco does (World Health Organization 2017b).

**Fig. 1:**
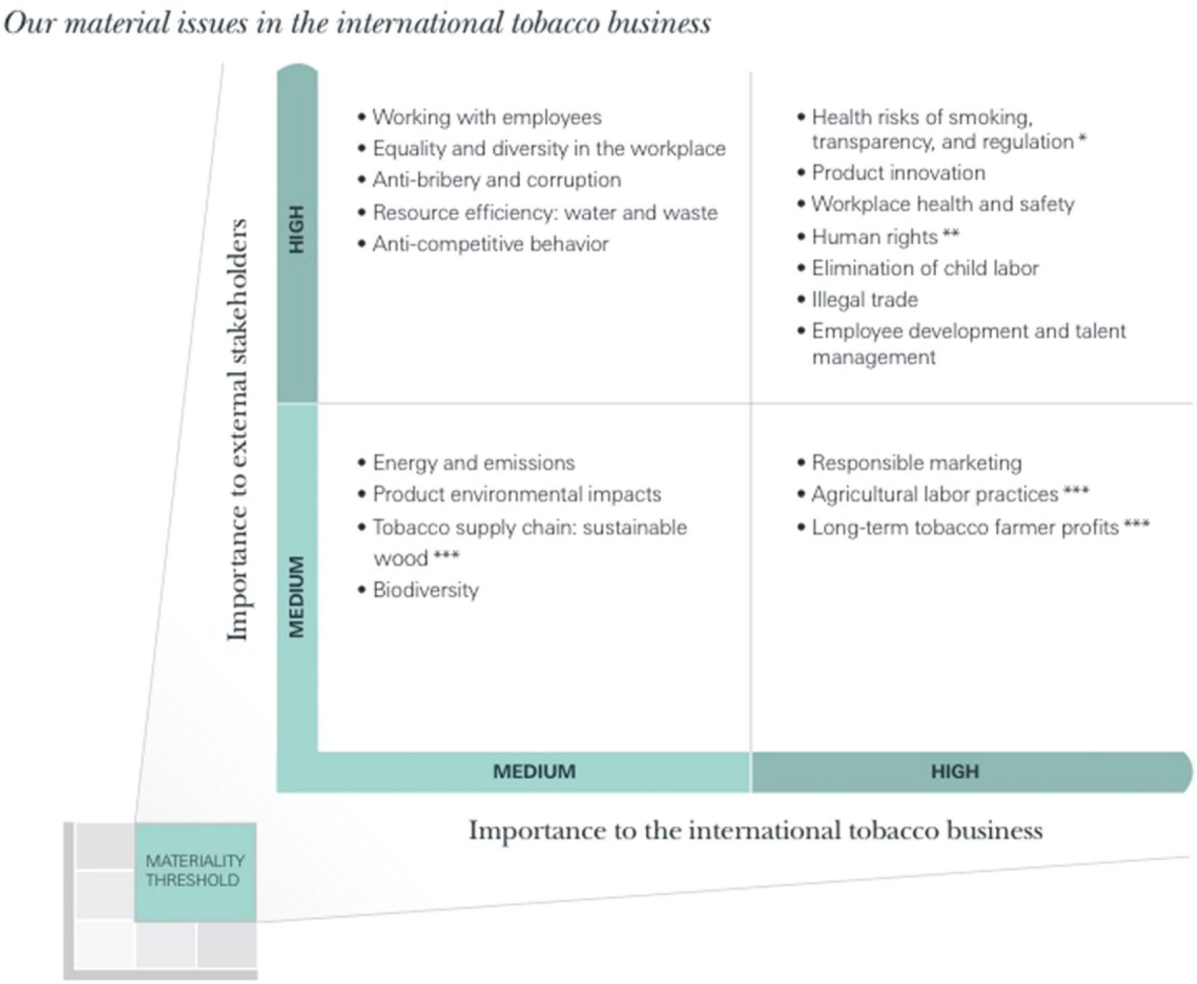
JTI’s graph of prioritization (Japan Tobacco Incorporated 2015)

Tobacco companies appear to place the environmental externalities and global environmental impact of their business lowest in their list of priorities (Fig. 1), overlooking that the environmental costs of tobacco manufacturing and distribution extends beyond these companies. This may be due to fiduciary responsibilities, or a lack of research and awareness of tobacco’s environmental harms. The latter reason is supported by the fact that the Framework Convention Alliance (FCA), an umbrella group of tobacco control NGOs supporting the FCTC, in a literature review on each of the FCTC’s 38 articles, could not identify any literature on tobacco and the sustainable management of water and energy for their 2015 data report (Framework Convention Alliance and Campaign for Tobacco-Free Kids 2015). The FCA’s inability to locate relevant studies on the sustainability of the tobacco manufacturing reveals the need for systematic and independently verified data.

Industry estimations regarding what constitutes an environmental issue versus appraisals by regulatory bodies also diverge. In a reporting questionnaire from the Carbon Disclosure Project (CDP) asking “Was your organization subject to any penalties, fines and/or enforcement orders for breaches of abstraction licenses, discharge consents or other water and wastewater-related regulations in the reporting year?” PMI answered, “Yes, not significant,” while reporting that 10% of their facilities were cited and fined for wastewater violations. One of the violations was for sub-par wastewater quality, including “increased levels of detergents, phosphates, [and] ammonium nitrogen above relatively tight limits for these substances in the Ukraine” (Philip Morris International 2017a). PMI’s deemphasizing evaluation of the severity of their own violations, indicative of the industry as a whole, highlights a discrepancy between what qualifies as significant environmental health trespasses for the industry versus the determined limits of existing environmental health standards. There could be other environmental health violations that are not reported either because they are not regarded by the industry as violations or because such reporting is not required.

The UN Sustainable Development Goals (SDG) Article 12.4 specifically refers to the 2020 goal of achieving “the environmentally sound management of chemicals and all wastes throughout their life cycle,” while Article 3a explicitly calls to “[s]trengthen the implementation of the WHO Framework Convention on Tobacco Control in all countries, as appropriate” (United Nations 2015b). This heralds recognition of the crosscutting problem of tobacco on both human health and the environment.

The United Nations Environmental Program’s The Economics of Ecosystems and Biodiversity (TEEB) program’s report, *Natural Capital at Risk*—*The Top 100 Externalities of Business* found that if the major industries, including tobacco manufacturing, accounted for their unaccounted environmental impacts—38% which are greenhouse gas emissions, 25% water use, 24% land use, and 7% air pollution—they would not be profitable (Trucost and TEEB for Business Coalition 2013).

One problem with sustainability goals—both public and private—is the perennial problem of the shifting baseline (i.e., Pauly 1995). Percent reductions of emissions are always measured against a set date when emissions were estimated. If the baseline is the highpoint for polluting and inefficiency, then any improvement will appear a major gain. If, however, a previous or future baseline is taken, the same change over a different period might be cast in a less favorable light. Similarly, if the baseline is pegged at the height of cigarette production, and then fewer cigarettes are subsequently produced and sold, absolute numbers of water use and CO_2_ emission will appear to go down, but their actual efficiency (or intensity) may remain unchanged.

Another problem with voluntary environmental targets is that if a company fails to make the target, it is easy to simply stop reporting on the target or stop referring to the goal. One example is in BAT’s 2016 Sustainability Report. BAT held a “long-term standard” for “BAT-owned leaf suppliers to use no more than an average of 1.5 kg of active chemicals per hectare of tobacco per year” (British American Tobacco 2017). When in 2016 the average use of active chemicals per hectare of tobacco exceeded 2.16 kgs, BAT decided to “no longer have a global average target” and instead “will continue to work with our leaf suppliers to better understand how improvements in best practice can be applied in this area” (British American Tobacco 2017). The move from measurable, quantifiable environmental goals to less measurable data when targets are not met is indicative of the problems with voluntary, non-mandatory environmental initiatives.

A further problem generated by environmental goals in response to CSR positioning is the tendency to initiate more environmentally friendly practices in countries with environmentally demanding publics, while continuing lower environmental standards in facilities off the radar of environmental advocates. PMI’s flagship green factories are in developed countries rather than facilities in low- and middle-income countries (LMICs). They prominently display in their Sustainability Report that their “German factories are powered by electricity generated by 100% renewable sources” and that their “Canadian facilities in Quebec and Brampton reduced their energy consumption by over 10% through initiatives including [a] new building management system, upgraded boilers and energy efficient chillers” (PMI 2018). Imperial Tobacco likewise emphasized the energy efficiency improvements to their German factories, while remaining silent on plants in LMICs (Imperial Brands PLC 2017). These efforts to address point-source complaints often do not result in thoroughgoing environmental reforms and improvements at all facilities in countries where TCs command more economic leverage. The environmental costs of tobacco manufacturing present unaddressed environmental justice dimensions.

## Findings

The tobacco industry identifies manufacturing as the most environment-destroying step of tobacco production. Forty-three cents out of every dollar of industry costs goes towards the manufacturing process, in contrast to only four cents spent on purchasing tobacco leaf itself (Eriksen et al. 2015). A CSR report from Imperial Brands states, *“*Our greatest direct impact on the environment comes from our product manufacturing activities” (Imperial Tobacco 2006). As the ecological footprint from farming tobacco has been more completely assessed than manufacturing and has proven significant (Lecours et al. 2012), Imperial’s statement—and the likelihood that their disclosure reflects proportional ecological footprints of other tobacco companies—emphasizes the need to learn more about the environmental impact of tobacco manufacturing.

### Environmental impact components

Common environmental impacts on which tobacco companies (TCs) report include annual CO_2_-equivalent emissions, energy use and mix, water use, waste water effluent, tonnage of solid waste to landfill, percentage of waste recycled, and tonnage of hazardous waste. This is standard for most manufactures of products. The categories of reporting, however, were incomplete in the early 2000s, mostly focusing on complying with ISO 14001 and 14064 requirements related to environmental management in compliance with quantifying and reporting greenhouse gas (GHG) emissions and reductions (Delmas and Montes-Sancho 2011; Perego and Kolk 2012; British American Tobacco 2015). Self-reporting in the past decade has grown to include elaborate environmental audits by third-party certification consultants, including ascertaining some of suppliers’ environmental externalities along the commodity chain. Baselines established a decade ago by the industry itself become references for the industry to set benchmarks for more efficient processes, measured by decreasing inputs and externalities (e.g., CO_2_-equivalent emissions) to achieve a higher manufacturing *intensity* (or efficiency) per million cigarettes produced or per million dollars of revenue.

**Fig. 2:**
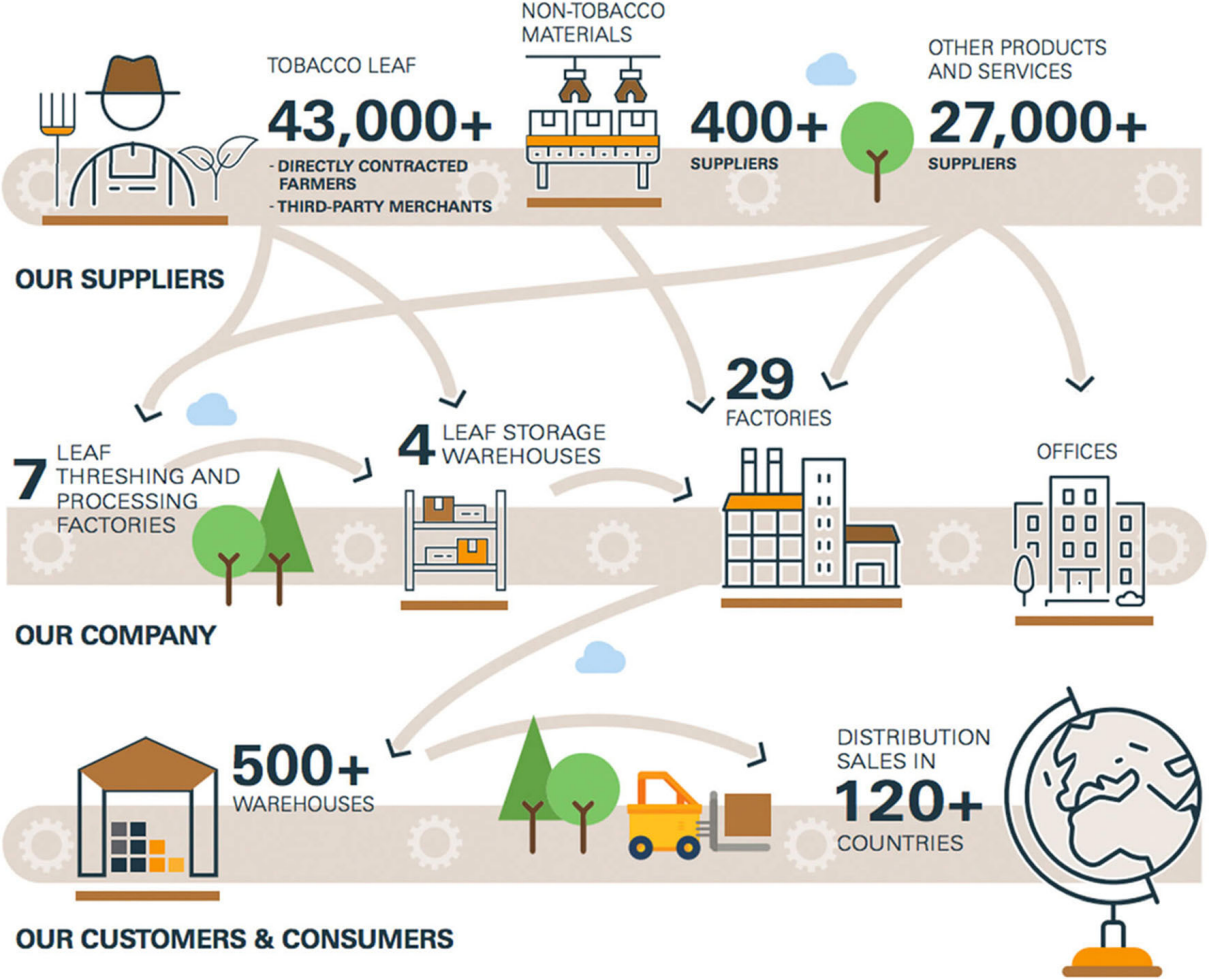
JTI supply chain diagram (JT Group 2017)

Reducing environmental harms from tobacco manufacture requires assessing all the primary points of pollution. Stanford University’s *Citadels* industry manufacturing facilities map (https://web.stanford.edu/group/tobaccoprv/cgi-bin/map/) provides insight into the scope of pollution caused by the 560 tobacco processing and manufacturing facilities worldwide. Various elements to tobacco manufacturing create waste and emissions, including preparation and treatment of the tobacco leaf, chemical additives, paper wrapping, filters, and other components, each demanding energy, water, waste, and materials. While there are many points of intervention in the tobacco product supply chain (Fig. 2), the leaf threshing and processing factories, storage, and warehouses—the components of tobacco manufacturing—are the aspects of the commodity chain best captured by current reported data.

### CO_2_-equivalent emissions

For CO_2_-equivalent (CO_2_e) emissions, the majority of release happens in the agricultural production of tobacco leaf, followed by the supply of non-tobacco materials and distribution and logistics (BAT 2015). Nonetheless, manufacturing pollution, distribution, and logistics (transport) pollution still comprise approximately a third of tobacco’s environmental impact due to CO_2_e pollution (Table 1).

**Table 1:** Reported CO_2_e emissions from tobacco manufacturing

Company; year reported; scope, if reported)	Thousands of tons CO_2_e	Tons per million cigarettes/dollars/pounds in revenue
Altria (2016) (Altria 2017)	**392** (total)	Not reported
	170 (scope 1)	
	210 (scope 2)	
	11 (scope 3)	
BAT (2016) (British American Tobacco 2016)	**862** (total)	0.81 per million cigs
	687 (scope 1-2)	
Imperial (2016) (Imperial Brands PLC 2017)	**329** (total: differs from categories below)	41.7 per £million
	26.7 (scope 1)	
	1.4 (scope 2)	
	187.6 (scope 3)	
JTI (2016) (Japan Tobacco Incorporated 2017)	**6513** (total)	0.65 per million cigs
	714 (scope 1 and 2)	
PMI (2015/16)	**5690** (total) (2016) (PMI 2017b)	0.66 per million cigs (2015) (PMI 2018)
RAI 2015 (RAI 2017)	**244** (total)	22.84 per US $million
Global total	**30 958 904**	

Bold numbers are total emissions (scopes 1 to 3 inclusive)

**Fig. 3:**
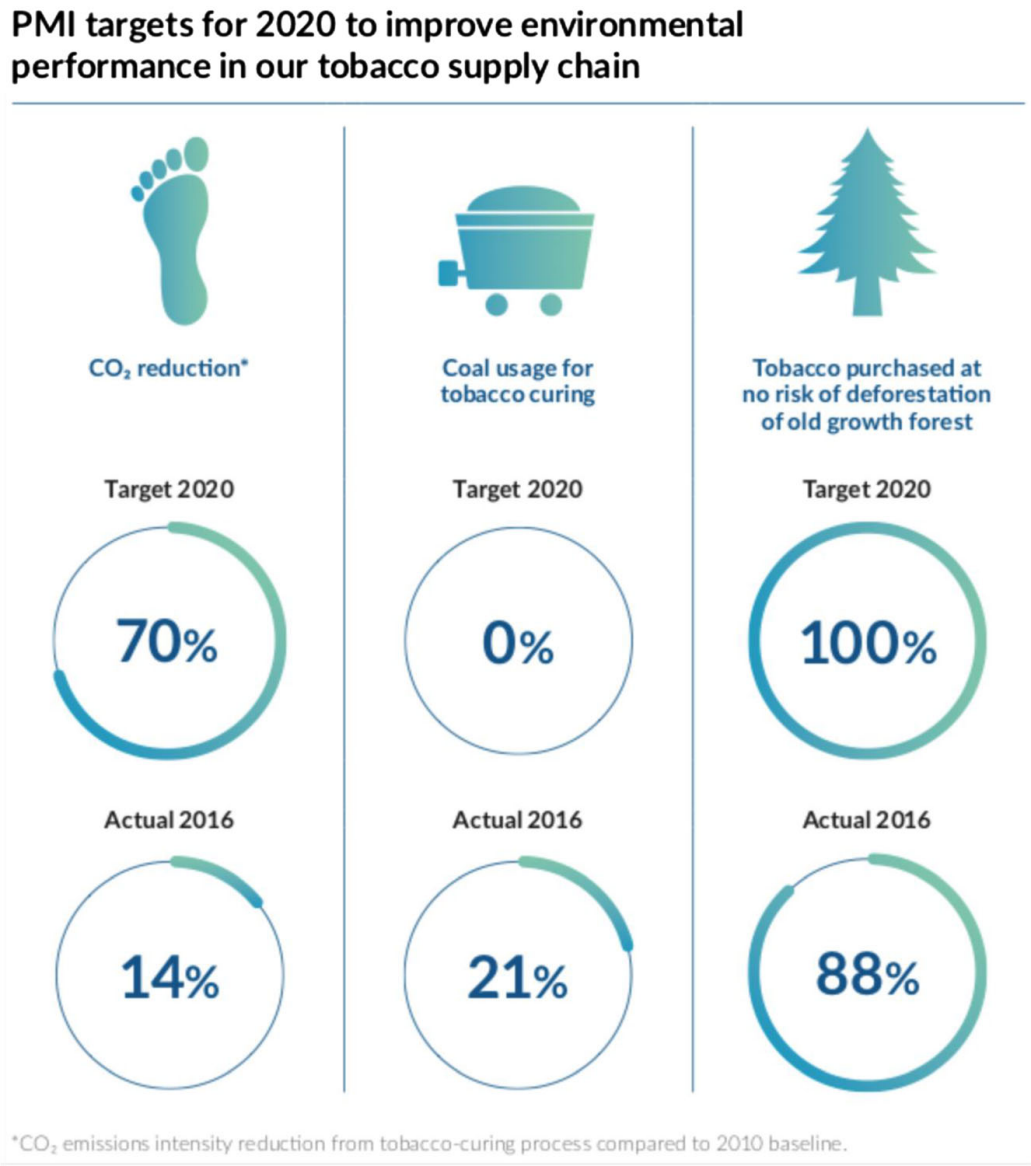
PMI Targets. From the PMI 2016 “Communication on Progress” for the UN Global Compact Report (Philip Morris International 2017b). Note the large gap between 2020 targets on reducing CO_2_e from the 2010 baseline and 2016 progress

To determine total CO_2_e emissions and other environmental harms, generally climate change policymakers distinguish between three different “scopes” of emissions and resource usage. Scope 1 emissions are direct emissions from sources directly controlled by a company or organization. Scope 2 emissions encompass emissions from energy use dependent on source type. Scope 3 includes indirect emissions, or CO_2_e embedded in purchased goods and services, transportation and distribution, capital goods, and activities not directly under the company’s control but which they can influence (Fig. 3).

For 2017 scope 1 emissions globally, for example, PMI emitted 229 116 tons CO_2_e from manufacturing, 118 487 tons due to its vehicle fleet, 3947 from aircraft, and 440 tons from its office activity. For scope 2 emissions, PMI emitted 434 460 tons CO_2_e from manufacturing, and 15 800 from offices. Included in these scope 2 emissions, PMI burned 250 645 megawatt hours (MWh) of diesel, 260 866 MWh of gasoline, and 41 348 MWh of brown coal, for a total of 923 345 MWh. Scope 3 CO_2_e emissions for PMI, however, reached 3 611 000 tons, their majority. These emissions include the carbon costs of burning wood and coal to cure tobacco as well as the materials for the cigarette such as packaging, cigarette papers, and acetate tow for filters (PMI 2017b) (Table 1). While PMI and other companies described instituting measures to reduce the most polluting types of energy use, such as replacing wood for curing with gas facilities, these interventions did not significantly decrease their emissions year-on-year.

Emissions by the global tobacco industry are roughly on par with those of other major industries. For comparison, the coffee house chain Starbucks, with 16 000 stores in 61 countries serving 50 million customers per week, emits 1 340 000 tons of CO_2_e per year (scope 1 & 2 in 2015) (Starbucks Coffee Company 2018) to PMI’s reported 1 150 000 tons for scope 1 and 2 (PMI 2017b). By extrapolation, assuming that other tobacco manufactures have similar greenhouse gas effluent, since PMI has 14.6% of the global tobacco market (Felsted 2016), the global total for tobacco CO_2_e emissions (scopes 1–3) is estimated to be 31 million tons of CO_2_e—about half Chevron’s 66 million tons CO_2_e 2016 emissions (Chevron Corporation 2017). By another calculation, the entire product lifecycle of a single cigarette contributes 5.72 grams of CO_2_e (Qian et al. 2016), leading to 39.4 million tons of CO_2_e for the 6.25 trillion cigarettes produced worldwide. That the tobacco industry’s CO_2_e emissions are in the same general category with a major oil company, without providing any social benefit, raises the social question of whether such continued emissions are worth their costs in exacerbating climate change.

### Energy use

As with CO_2_e emissions, with energy, companies make green claims as well, that they are decreasing scope 1 and 2 emissions. For example, in their 2014 CSR Report, Altria states that it “converted coal-fired boilers to natural gas boilers at three manufacturing facilities, significantly decreasing Scope 1 greenhouse gas emissions” (Altria 2015). BAT derived nearly all of its energy from non-renewable sources (British American Tobacco 2018). PMI’s reported energy use is anomalously low, less than half of that of BAT, even though PMI produces more cigarettes worldwide. For this reason, BAT’s energy use has been used here to extrapolate total global total energy usage, as BAT is between JTI and PMI both in terms of cigarettes produced and total energy use (Table 2).

**Table 2:** Reported yearly energy use for some of the largest tobacco companies

Company	Gigawatt hours/year	Kilowatt hours per million cigarettes/$/£ revenue
Altria (2016) (Altria 2017)	1316 (Altria 2017)	Not reported
BAT (2016) (British American Tobacco 2018)	2360 (276 renewable)	2911 per million cigs
Imperial (2016) (Imperial Brands 2017)	880	137 664 per £million
JTI (Japan Tobacco Incorporated 2017)	2632 (2016) (665 renewable)	Not reported
PMI	923 (2017) (PMI 2017b)	107 500 per US$ million (2012) (Philip Morris International 2017b)
RAI (2015) (Reynolds American International 2015)	904	84 639 per US$ million
Global total	16 164	

All major tobacco companies consume various forms of fossil fuels. In terms of the mix of energy consumed in 2016, just counting nonrenewable resources, Altria, for instance, consumed 22.6 million hundred cubic feet (hcf) of natural gas, 36 176 gallons of fuel oil, 870 293 gallons of propane, 151 743 gallons of diesel, 2 789 801 gallons of gasoline, and 429 381 gallons of jet fuel (Altria 2017). But what is not included in Altria’s report is that a Trucost report found that “Tobacco company Altria Group Inc, the parent company of Philip Morris USA, has the highest carbon intensity in the [entire] Personal and Household Goods sector,” placing Altria in the same carbon intensity group as oil and coal companies, the highest quintile (Trucost 2009). While Trucost focused on Altria in their report, the company is not especially anomalous among major tobacco companies for their high use of carbon-intensive fuels.

### Intensity

Manufacturing *intensity* refers to how much per unit of product is required for a given metric, such as energy, CO_2_ emissions, water use, or waste production (Japan Tobacco Incorporated 2017). For example, compared to their 2009 baseline, in 2013 JTI required per cigarette roughly 10% more energy, 5% more CO_2_e emissions, but 10% less water. Their report did not contain the raw data, however. Reporting in per million cigarettes only, instead of also including absolute numbers, obscures rising overall environmental costs, as the company produces more cigarettes each year. Even if manufacturing becomes more efficient for some measures, if more total cigarettes are produced, environmental harm is nonetheless increased. While during the 2000s and early 2010s the standard unit of measurement for intensity was “x amount of [water, CO_2_, energy, etc.] per million cigarettes produced,” a recent trend has been to not mention the amount of environmental impact per cigarettes produced by instead measuring intensity in environmental costs per million of US dollars or British pounds of net tobacco revenue (Imperial Tobacco Group PLC 2015).

### Water consumption and discharge

Tobacco manufacturing is extremely water-use intensive for plant commodities (Table 3). While TCs claim incremental gains in water conservation over previous years, their impact on freshwater remains substantial. In the available data, Altria’s water consumption reporting is anomalously high. Imperial acknowledges that 92% of all water use occurs in tobacco growing, with another 7% used in paper and cardboard manufacturing, with only 1% of their water use due to end-product manufacturing (Imperial Tobacco Group 2014). Using contracted but non-company suppliers for their tobacco leaf and other raw materials, TCs can omit these environmental impacts from their public sustainability reporting, even if they privately hold full life cycle analysis data.

**Table 3:** Reported water consumption used during tobacco products manufacturing

Company	Thousands of cubic meters	Cubic meters per million cigs produced/£/$ million revenue
Altria (2016) (Altria 2017)	9422	Not reported
BAT (2017) (British American Tobacco 2018)	3667	3.43 per million cigs
Imperial (2016) (Imperial Brands 2017)	1648	230 per £million
JTI (2016) (Japan Tobacco Incorporated 2017)	9896	Not reported
PMI (2016) (Philip Morris International 2016)	3394	3.95 per million cigs
RAI 2015 (Reynolds American International 2015)	1898	177.75 per US$ million
Global total	23 247	

Companies report less transparently on the amount of water they discharge, the refuse water released into the environment resulting from the manufacturing process (Table 4). Some companies, such as BAT which claims to recycle and reuse 11% of its wastewater (British American Tobacco 2018), aim to recapture their wastewater to reduce freshwater usage and the contamination problems waste water presents.

**Table 4:** Reported water discharge during tobacco product manufacturing

Company	Thousands of cubic meters	Per million cigs (cubic meters)
Altria	Not reported	Not reported
BAT (British American Tobacco 2018)	2156 Total as sewage: 2108	2.01 per million cigs
Imperial	Not reported	Not reported
JTI (JT Group 2018)	5527	Not reported
PMI (2016) (PMI 2017b)	1901	Not reported
RAI (2015) (Reynolds American International 2018)	1898	130 per US$ million
Global total	13 021	

### Waste disposal: Landfill, recycled, hazardous waste

#### Waste disposal: Landfill

For manufacturing, the sources of waste are both tobacco and constituents (Table 5). JTI, for example, purchases annually over 300 000 tons of non-tobacco materials for processing, much of which ends up in landfills after use (Japan Tobacco Incorporated 2013). JTI also reported in 2016 that 77% of waste is recycled, and 8% recovered, with 15% ending up in the landfill (Japan Tobacco Incorporated 2017).

**Table 5:** Reported waste disposal related to tobacco manufacturing: Landfill

Company	Millions of Pounds	Tons per million cigs/per $/£ million revenue
Altria (FY 2014) (Altria 2015)	22.7	Not reported
BAT (2016) (British American Tobacco 2018)	287	0.12 metric tons
Imperial (2015/16)	109.6 (Imperial Brands PLC 2017)	1.4 per £million (Imperial Tobacco Group 2015)
JTI (2016)	25 (Japan Tobacco Incorporated 2017)	0.17 per million cigs (Japan Tobacco Incorporated 2013)
PMI (2015)	280	Not reported
RAI (2015) (Reynolds American International 2018)	56.6	2.4 per million dollars
Global total	1917	

#### Waste disposal: Recycled

While all companies report on their total waste, fewer document the percent of waste they recycle from the manufacturing process. For some companies, it is unclear what type of handling of materials is included under the heading “recycled,” and how much environmental effect these efforts have, without a more detailed and transparent reporting concerning what recycling waste entails. For companies reporting waste recycling percentage, Altria reported 74.3 million pounds of recycled waste (Altria 2015); JTI recycled 78% of its waste (JT Group 2018); and RAI reported 69% of its solid waste is recycled (RAI 2017).

#### Waste disposal: Hazardous waste

According to the *Toxic Release Inventory Database*, over a million pounds of toxic chemicals were released in 2008 from tobacco manufacturing plants, including ammonia, nicotine, hydrochloric acid, methanol, and nitrates (The Right to Know Network 2008). In terms of specific reporting, in 2011 BAT reported that 1973 metric tons of hazardous waste were produced from the tobacco manufacturing process (British American Tobacco 2011); Altria discharged 999 lb of phosphorus in wastewater, and 17 000 lb of nitrogen, according to their 2014 CSR Report (Altria 2015); and Imperial produced 330 tons of hazardous waste in 2016 (Imperial Brands PLC 2017).

### Environmental manufacturing goals

Another aspect of TCs’ CSR programs is to establish ‘Environmental Goals’ for their manufacturing processes (e.g., PMI 2018). These include measurable reductions in energy use, increases in the proportion of facility waste that is recycled or reused, and reduced CO_2_e emissions and water consumption, among other common stated goals. For example, BAT’s 2014 sustainability report claimed a 45% reduction in CO_2_e emissions against 2000 emissions (British American Tobacco 2015), and other companies highlight what they are doing to mitigate greenhouse gas (GHG) emissions from their production facilities. Altria’s 2014 Environmental Manufacturing Goals for 2016 included reducing energy use by 10%, reducing GHG emissions by 20%, achieving 50% water neutrality (recycling water and investing in clean water elsewhere), recycling or reusing 95% of facility waste, and reducing packaging materials by 5 million pounds (Altria 2015). BAT emphasized its green credentials based on its inclusion in the Dow Jones Sustainability World and Europe Indexes in 2011 (British American Tobacco 2011). They claimed, “To reduce our carbon footprint, we address our energy use, our waste to landfill and our business travel. We are also beginning to explore opportunities for generating and purchasing renewable energy” (British American Tobacco 2011). At the same time, BAT reported that in addition to the 909 496 metric tons of tobacco leaf they used in their products, they also used 442 893 metric tons of other materials including cigarette paper, wrapping, packaging, filters, glues, and inks, plus 41 951 metric tons of indirect materials such as cleaning agents (British American Tobacco 2011). Industry-initiated environmental goals appear to be based on revenue-capturing low hanging fruit rather than actually substantially addressing the most severe environmental costs of business.

### China National Tobacco Company

Extrapolating from the industrial ecology self-reporting from the largest tobacco companies, a total environmental impact can be ascertained, even in the absence of publicly available data from the Chinese National Tobacco Company (CNTC). The CNTC has nominally expanded into markets outside China (1% of total sales); nonetheless it produces roughly 44% of the cigarettes consumed globally (2.5 trillion out of 6.25 trillion) (Euromonitor International 2016), with China consuming roughly ten times as many cigarettes as any other nation (Campaign for Tobacco-Free Kids 2017). Thus, without data from the CNTC, evaluating the global environmental impacts of TC manufacturing only accounts for roughly half the global total.

As a government-owned company, the CNTC does not have the same transnational shareholder demands for reporting environmental accounting, as limited as these are. What is known, is that CNTC disposes an estimated 175 000–600 000 cubic meters of wastewater per year, which contains fine suspended particles as well as aromatic compounds and nicotine (China Bike 2015). One source in the Chinese edition of *Fortune* magazine reports that for CNTC the “…total industrial emissions of sulfur dioxide [amount to] 5688 tons, down 29.8%; chemical oxygen demand emissions are 2751 tons, down 11.7%” (Xinhya News 2012). No baseline is given in the article. However, one CNTC subgroup, Jia Yao Holdings Limited, reported to have incurred environmental costs of approximately RMB451,000 (~ $70 000 US) for 2014 and RMB589000 (~ $90 000 US) for 2013, according to their annual report (Jia Yao Holdings Limited 2015). We were unable to determine whether these are government fines for polluting or other costs, and what share of market Jia Yao commands. Jia Yao purports to comply with China’s Law on the Prevention and Treatment of Solid Waste Pollution and Law of the People’s Republic of China on the Promotion of Clean Production. Such environmental claims, however, are undermined by statements such as “[t]he Directors are also of the view that our production process does not generate hazards that will cause any significant adverse impact on the environment” (Jia Yao Holdings Limited 2015); other transnational tobacco companies are very aware indeed of their environmental impact: hence their strenuous reported efforts to reduce their impact. Such appraisals of environmental impact are at odds with what is known about the environmental impacts of tobacco manufacturing as reported by other tobacco producers. While China grows most of the tobacco CNTC uses, it has started expanding into other areas, such as its recent use of Zimbabwe tobacco, where it recently also set up manufacturing facilities (Samukange 2015).

### Electronic cigarettes: A looming environmental threat

The rise of electronic cigarettes (e-cigarettes) in industrialized countries is changing the composition of the environmental harms of tobacco (Ligaya 2013; Chang 2014; World Health Organization 2017a; Hendlin 2018; PMI 2018). Because these products are composed of low-value but sophisticated electronics, the environmental costs from manufacturing e-cigarettes may be much more severe than cigarettes per unit (Hendlin 2018).

E-cigarettes made in different countries are manufactured according to the standards of the manufacturer’s country, and do not always conform to laws for exposures to metals and other toxins in the countries they are used. In the United States, e-cigarettes originally were to be included as drug-delivery devices under the US Food and Drug Administration, which would have required much stricter product regulation. However, a 2010 suit overturned this designation (Committee on the Review of the Health Effects of Electronic Nicotine Delivery Systems et al. 2018). The 2016 FDA Deeming Rule aimed to place e-cigarettes under a 2007 regulatory cut-off which would require extensive testing of e-cigarettes if they wished to remain on the market. As the deadline for this requirement has been postponed from 2018 to 2022, e-cigarette manufactures are free to produce and sell devices with minimal oversight by health or environmental regulatory institutions (Eilperin 2017). In the UK, while e-cigarettes disposal and reclamation must adhere to the Waste Electrical and Electronic Equipment Regulations, requiring companies to receive and process electronic waste (BAT 2018), the arduous process of sending these products back to manufactures and having to pack and pay for postage to responsibly return these products likely limits the effectiveness of such consumer-side responsibility to unknown efficacy.

The chemical content of e-liquids and the construction of e-cigarettes vary widely—from disposable single-use “cig-a-like” products resembling cigarettes, to refillable “vape pens,” to “mods” and “tanks.” The best-selling device in the US as of 2018 is the Juul cartridge-based or “pod” e-cigarette (Craver 2018). While the USB stick-shaped device is not single-use, its hard plastic e-juice cartridges are. Because of the overwhelming diversity of products, no blanket assertion on the environmental impact of these products is possible. Introducing new classes of plastics, metals, cartridges, lithium-ion batteries, and concentrated nicotine solutions, however, involves significantly more environmentally intensive manufacturing processes than products that are primarily made of plant material and plastic filters, as combustible cigarettes are (Goniewicz et al. 2013; Lerner et al. 2015).

Ibis World, an industry market research company, predicts that “the [traditional] Cigarette and Tobacco Product Manufacturing industry is in the declining stage of its life cycle” (IBIS World 2017). They note, however, that the industry will resist this decline through expansion into electronic cigarettes and other electronic nicotine delivery devices.

The tobacco industry is aware of the new scope of environmental harms e-cigarettes pose. PMI discussed the “need to manage new areas of impact due to the increasing use of electronics and batteries in our products” (Philip Morris International 2016). As tobacco companies increasingly are selling electronic smoking devices, they acknowledge that “while we embed new processes, the efficiency of our energy and water use may worsen until both knowledge and economies of scale improve” (Philip Morris International 2016). PMI’s Lifecycle Analysis (LCA) performed for e-cigarettes and other so-called reduced-risk products (RRPs) “highlighted the impact that RRPs will have in [their ecological] footprint and plans in product development, manufacturing, distribution and rest of value chain have been implemented to mitigate their impact in our footprint” (PMI 2017b).

Fundamentally, the tobacco industry has been aware of “cradle to grave” extended-producer responsibility manufacturing since at least as early as 1991 (GJW Government Relations 1991), and has nonetheless refrained from implementing practices that could reduce the waste from their products, both in terms of production and disposal. Conventional cigarette filters, for instance, have been proven to do more harm than good in terms of health (Song et al. 2017), and these unnecessary appendages to cigarettes, originally developed in the 1950’s to assuage growing fears over the health harms of cigarettes, directly harm the environment in their material production and disposal (Pollay and Dewhirst 2002; Smith and McDaniel 2011; Song et al. 2017). Based on reviewing industry documents, it does not appear as if any cradle-to-grave industrial ecology has been undertaken to minimize the amount of ecological impact of e-cigarette manufacture and disposal.

## Discussion

### Lack of standard reporting measures and independent third-party oversight

The impacts of tobacco manufacturing on ecosystems, humans, and animals are difficult to quantify. Under the guise of proprietary information, often rationalized to prevent counterfeit manufacturing, tobacco industry manufacturing processes are closely guarded secrets (Imperial Tobacco 2006); this proprietary protection further inhibits research into environmental impacts of the manufacturing process. Another concern with self-reported data is that not all manufacturing plants are considered in these reports. For example, for unknown reasons Imperial Tobacco omits data from their manufacturing facilities in Laos and Turkey (Imperial Tobacco Group 2015). Without including environmental costs into the actual sales price of tobacco products, governments inadvertently subsidize tobacco use and enable the tobacco industry to externalize the environmental costs of their products. Countries such as Brazil and Canada have mandated tobacco manufacturers to disclose information on manufacturing practices, product ingredients, toxic constituents, and toxic emissions to evaluate the environmental impacts of tobacco production in these countries (World Health Organization 2008). More stringent compliance is necessary globally, and while accurate disclosure can assist in mitigating obvious violations, this do not always translate into decreased emissions.

Voluntary initiatives, furthermore, can be interpreted in the literature as proactive moves by the industry to stave-off regulation which would require them to adhere to externally wrought environmental standards and practices (Soneryd and Uggla 2015).

### Tobacco company involvement in environmental and social stewardship promotion organizations

While tobacco industry environmental reporting remains fragmentary, previous industry involvement in the United Nations Global Compact (UNGC) has revealed finer-grained environmental impact data than their sustainability reports or annual reports. Thus, industry involvement in these organizations has motivated them to disclose more data regarding their real environmental harms, giving environmental scientists and industrial ecologists some data for analysis. At the same time, involvement in the UNGC and the Carbon Disclosure Project (CDP) lent a veneer of respectability and credibility that allows the industry to be seen more as “partners” in public health and environmental sustainability than their deserved reputation as sullying both. PMI, for example, praised:

We work on the UN Global Compact and have published our first communication on progress to the United Nations Global Compact, reporting comprehensively on our sustainability practices across human rights, labor rights, environment and anticorruption.… We are also part of the World Business Council for Sustainable Development (WBCSD), the WeMeanBusiness coalition, and since participating in the UNFCCC COP21 in Paris, we have continued to engage externally regarding our commitments on climate change adaptation and water, including our support for the Paris Agreement. (Philip Morris International 2017a).

Such credentials sound impressive, and constitute CSR virtue signaling. Therefore, including tobacco companies into organizations such as the UNGC, the UNFCCC or CDP may dilute the designation or brand of the conferring organization, while giving a false sense of achievement to the company, that it can then parade to the public. As of October 15, 2017, however, as part of an integrity review, the UN Global Compact no longer allows tobacco companies to be part of the initiative (van der Eijk et al. 2017), and thus PMI and other tobacco companies can no longer claim their mantle of support. Whether other organizations follow suit, such as the Carbon Disclosure Project, remains to be seen. Additionally, the cost of false credibility must be weighed against the detail of reporting. If these business recognition organizations extract more accurate and precise data from the companies—which can be debated—then they certainly have some merit, despite their social and political enablement. Instead of trading data for legitimacy, governments could mandate the industry to disclose third-party verified data, setting goals to reduce environmental harms.

### Ecological modernization and greenwashing

**Fig. 4:**
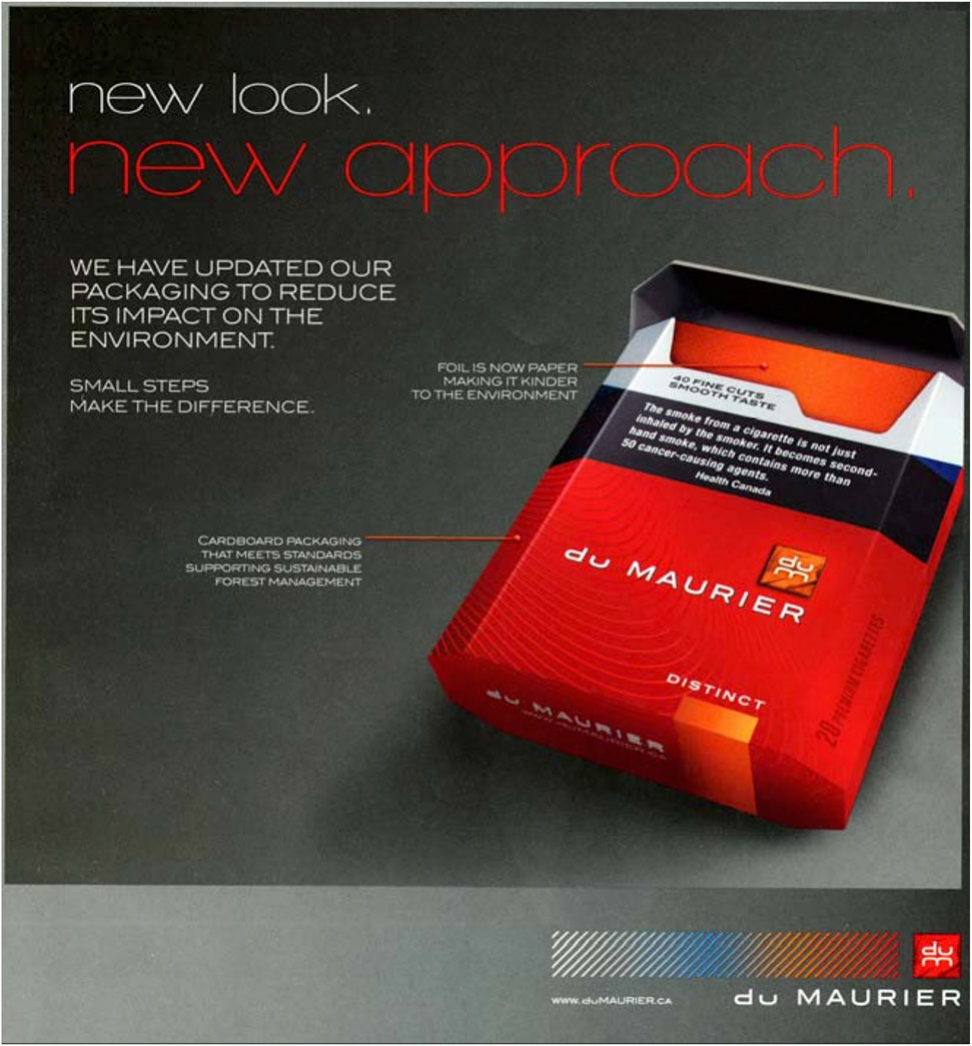
Foil-free, plastic-free, sustainably-managed cardboard cigarettes (2009, BAT-owned Canadian du Maurier brand) (Steeman 2009)

One important consideration is the overall sustainability of the tobacco industry in general. A 2004 WHO report called tobacco industry CSR an “inherent contradiction” (World Health Organization 2004). While the issue of increasing efficiency of manufacturing and transport processes to decrease the ecological harms by the industry is real, it cannot be ignored that industrial tobacco manufacturing is a polluting process producing a hazardous product with adverse environmental impacts and justice concerns. Manufactures have been aware that consumer perceptions of their manufacturing processes have been scrutinized, and are trying to allay such concerns. For example, BAT (Canada) created biodegradable packaging and more ecological manufacturing practices as selling points for their popular brand of cigarettes (Fig. 4); others, such as RJ Reynolds have emphasized investments in “green transport” (RAI 2017).

Although CSR reports highlight sustainability initiatives by the TCs, actual environmental impacts of manufacturing and transport remain a low priority for TCs (Reynolds American International 2018), and a low priority to date for tobacco control advocates (Framework Convention Alliance and Campaign for Tobacco-Free Kids 2015). However, the inclusion of the WHO FCTC in the UN Sustainable Development Goals and the WHO FCTC Conference of the Parties’ decision on improving the understanding of the environmental impact of tobacco indicates that these issues will gain higher visibility and priority.

Some TCs advertise buying carbon offsets for portions of their emissions, and increasingly show interest in the money-saving aspects of reducing their energy footprint and increasing their efficiency (PMI 2017a). At the same time, it appears that carbon credits for factories in the EU are taken advantage of when they are convenient to the businesses (i.e., low cost), but are not maintained in other markets. PMI also noted that “Regulations requiring carbon labelling on products could impact PMI for both conventional cigarettes and our Reduced-Risk Products (RRPs) [such as e-cigarettes], which may include electronic components” (PMI 2017b). They note that the impact of carbon labelling on their different (conventional versus electronic) tobacco products “could also be an opportunity for PMI,” if they are able to differentiate themselves with low-carbon products vis-à-vis their competitors.

### Regulation rather than voluntary CSR

Rather than exhibiting authentic corporate responsibility, TC manufacturing activities comprise a hodge-podge of voluntary measures aimed at staving-off regulation (Palazzo and Richter 2005). The tobacco industry is known for moving from countries to avoid facing the consequences of their activities, including environmental harms (Gilmore 2004; Benson 2011). In 2013, after neighborhood leaders near the BAT Ugandan plant complained of fouled air, and the Parliament moved to draft a law more strictly regulating the production and sale of tobacco in the country, BAT closed their Ugandan plant and moved these facilities to Kenya (Wesonga and Butagira 2013). When citizens petition for better business or environmental practices, TCs (and other polluting industries) routinely uproot their operations and take them where civil society has less political influence and where fewer regulatory controls on manufacturing exist. Such actions undermine the plausibility that the greening of the tobacco industry springs from altruistic or environmental concerns, rather than public pressure, preempting government regulation, and cost-saving measures.

At the same time that TCs sometimes evade regulation in regions overlooked by global public health environmental advocacy, TCs respond to public outcry and pressure orchestrated in developed countries. In countries where environmental sustainability is an important political agenda item, TCs prioritize ecological modernization—the process of rationalizing production to save money while adopting greener technologies (Hajer 1996). In countries with less oversight, such actions are absent. Holding the tobacco industry accountable everywhere for the environmental justice externalities of the manufacturing and transport of tobacco, measurable by a variety of environmental indicators, is crucial to achieve continued reductions in TCs’ ecological footprint and a fair assessment of the product’s true cost.

### Voluntary life-cycle assessments (LCA) versus mandatory extended producer responsibility (EPR)

Extended producer responsibility (EPR) programs and legislation could require the tobacco industry to pay for take-back programs and incentives that help to keep tobacco product waste out of the environment (Novotny et al. 2015). Such programs would be managed by government agencies and other non-profit organizations, carried out independently from the tobacco industry, and could promote awareness campaigns regarding the human and environmental toxicity of tobacco product waste.

To preempt regulation, PMI has begun investigating the efficacy of life-cycle assessments, which might sidestep pressure for third-party analysis and interventions (Philip Morris International 2017b). This strategy of preempting policy intervention through undertaking voluntary reporting and self-censure has been used previously by the industry (McDaniel and Malone 2009; McDaniel et al. 2016, 2018). PMI’s performance of LCAs may indicate their awareness that LCAs are used for EPR, and could be used to preempt EPR regulation. EPR for the environmental costs of tobacco has been proposed by the European Union Commission as a potential solution to the tobacco epidemic:

One very straightforward solution which the consulting group suggested was to calculate the extra cost of smoking—hospital admissions, days lost to work, litter clean up and so on—and then to charge this to the tobacco companies on a pro rata basis according to market share. Once a year, Philip Morris et al. would get a bill for their share of billions of Euros that these externalities comprise. (Hastings 2012, p. 179)

There is no reason why such an EPR framework could not be applied to the harms to the environment. Especially for “luxury emissions” (Shue 1993), as tobacco products uncontroversially are, these emissions should be taxed according to their total harms.

## Limitations

The data in this review were limited to partial reporting by the tobacco companies. The opacity of self-report data regarding the actual environmental input and output of tobacco manufacturing serves as a major barrier to objectively evaluating the true environmental costs of tobacco production. Missing data, inconsistency of reporting across companies, uneven reporting on production intensity, and problems of transparency and reliability remain. The contrasting metrics different companies and even the same company in different years use in self-reporting (i.e., liters versus gallons), hinder comparative evaluation of resource use and effluence between companies. Also challenging, is that definitions of manufacturing intensity are not standardized. Some companies report efficiency or intensity per million cigarettes produced, while others adopt measures per million dollars/pounds in revenue, providing no common unit for analysis, complicating comparisons across companies.

Because the environmental impacts of tobacco manufacturing are not independently regulated and monitored, little has been reported outside of the industry’s own analyses. Without a stable, historical, or uniform baseline, global projections can only be extrapolated from existing industry data. Additionally, company-wide self-reported data from China’s National Tobacco Company (CNTC), if publicly available, were not locatable by us, even by native language research assistants. At best, we can assume that a company as large as CNTC is no less polluting, inferring from other Chinese manufacturing processes (Pratt 2011; Liu et al. 2016). The result is that the estimates made here through extrapolation likely severely underestimate the real environmental costs of global tobacco manufacturing.

The focus of this analysis was mainly cigarette manufacturing. While cigarettes still comprise almost 90% of all tobacco sales globally (except for South Asia), other tobacco products, especially e-cigarettes, also weigh heavily on the environment (Eriksen et al. 2015).

## Conclusion

The actual environmental impact of tobacco manufacturing remains unknown. Publically available data are selectively self-reported by the tobacco industry, and measured through accounting and consulting firms that have a direct interest in maintaining positive relationships with the tobacco companies funding them. As such, reporting may be opportunistic both in the scope of data reported and presentation, highlighting sustainability success while omitting data on environmental damages or increased emissions due to manufacturing that do not hew to the desired progressive narrative arc of reducing ecological externalities. This piecemeal reporting—rife in CSR reports across industries (Gray 2010; Perego and Kolk 2012), but especially trenchant for an industry with decades of documented manipulation of public opinion and science (Michaels 2008; Oreskes and Conway 2011; Proctor 2012)—raises serious doubts regarding the tobacco companies’ commitments to reducing the environmental consequences of tobacco manufacturing.

As the 2017 WHO report on the environmental impact of tobacco concludes, “the adage ‘there is no such thing as a safe cigarette’ could be extended to assert that there is no such thing as an environmentally neutral tobacco industry” (World Health Organization 2017a). Especially, if these companies adhered to Trucost accounting which incorporates environmental externalities (water use, air pollution, land degradation, etc.) (Trucost and TEEB for Business Coalition 2013), tobacco would not be a profitable industry. Yet, until the tobacco industry is required to internalize its social and environmental harms, citizens, governments, future generations, and the earth are subsidizing the profits these companies reap. While for some products this trade-off may be judged acceptable in exchange for the goods an industry provides to society, tobacco provides no such social good, and deserves a utility calculus accounting for all of its ranging harms, including environmental ones. Parties’ implementing the WHO FCTC should consider the environmental impact of tobacco product manufacturing and transport within the context of implementing Article 18 and expand the current focus on tobacco growing to a more comprehensive environmental approach. Countries striving to reach the SDGs by 2030 must incorporate the environmental harms of tobacco as part of their strategies to reach these goals, adopting regulations mandating extended producer responsibility.
